# Yeasts and bacteria associated with kocho, an Ethiopian fermented food produced from enset (*Ensete ventricosum*)

**DOI:** 10.1007/s10482-018-1192-8

**Published:** 2018-10-27

**Authors:** Genet Birmeta, Albina Bakeeva, Volkmar Passoth

**Affiliations:** 1 Institute of Biotechnology, Addis Ababa University, P. O. Box 1176, Addis Ababa, Ethiopia; 2 Department of Molecular Sciences, Swedish University of Agricultural Sciences, Box 7015, 75007 Uppsala, Sweden

**Keywords:** *Ensete ventricosum* (enset), Food fermentation, Food spoilage, Kocho

## Abstract

Enset (*Ensete ventricosum)* is the basis of the staple food consumed by about 20% of the Ethiopian population. Kocho is one of the food products generated from enset by spontaneous fermentation of decorticated and pulverized pseudostem and corm sections. We isolated culturable microbes associated with kocho from different stages of fermentation. Twelve yeast species, six lactic acid bacteria (LABs) species and eleven species of aerobic bacteria were identified by sequencing ITS/D1D2 regions of 26S rDNA of yeasts and 16S rDNA of bacteria, respectively. More yeast species were identified in fresh (fermented for 2–5 days) kocho, compared to long-term (7–12 months) fermented kocho, while we observed an opposite trend for LABs. In fresh kocho, the most frequently isolated yeast species were *Pichia exigua*, *Galactomyces geotrichum*, and *Pichia fermentans*. From mid-term (3–4 months) kocho most frequently *Candida cabralensis*, *G. geotrichum*, and *Candida ethanolica* were isolated. In the long-term fermentations, the most frequently isolated yeast was *Saturnispora silva*. *Lactobacillus plantarum* was the most frequently isolated LAB in both fresh and mid-term kocho. In long-term fermented kocho, *Acetobacter pasteurianus* and *L. plantarum* were most frequently isolated. *L. plantarum* was consistently isolated from all the three stages of fermentation. Aerobic bacteria in fresh kocho were mostly gram-negative, with *Raoultella planticola* and *Pantoea agglomerans* being the most frequently isolated species. In long-term fermented kocho, mainly gram-positive, spore-forming bacteria of the genus *Bacillus* were found, among them also species of the *Bacillus cereus* group, *Bacillus anthracis* and *Bacillus thurigiensis*.

## Introduction

Enset or false banana (*Ensete ventricosum* (Welw.) Chessman) is a staple food crop used by about 20% of Ethiopian population. Due to its relative tolerance against drought, heavy rains and flooding, enset has the potential to ensure nourishment of the Ethiopian population even at extreme weather conditions. Due to its multi-purpose application potential and its robustness there is a potential to expand its production to other regions of Ethiopia and other African countries. Enset food can be harvested at any time when other crops fail to grow due to drought. Furthermore, it can be stored for longer period and thus is available all year round (Birmeta et al. 2002, 2004a; Daba and Shigeta 2016).

Enset food products are traditionally fermented using the natural microbial flora. Little is known about the microbial flora associated with enset, thus the processes involved in production of the traditional fermented starchy foods (kocho, bulla) are not well understood. In a previous study, we demonstrated that enset supports the growth of several yeast and bacterial species (Birmeta et al. 2004b).

Kocho, an acidic starchy food is produced from the inner, non-pigmented portion of the trunk, pseudostem and corm. The material is decorticated, pulverised, mixed and kneaded into a mash. This mash is covered with layers of enset leaves and left at ambient temperatures (22–26 °C) for 2–5 days. Thereafter, the mash is filled into a pit, often one meter underground, which is covered with layers of enset leaves. Thereafter the pit is covered with soil and pressed tightly. Occasionally, the mash is taken out from the pit, mixed carefully, the old leafs are replaced and the mash is put back to the pit. This procedure may be repeated until consumption of the mash. During handling of the mash, spontaneous fermentation occurs. Fermentation times vary from few weeks to several months or years. Kocho qualities, such as taste, colour (discolouration), texture, or aroma, are often compromised due to the action of some microbes in particular when they proliferate under favourable conditions. Furthermore, the fermentation of kocho, involving yeasts and LABs, has a major influence on the nutritional and food quality of enset food products (Bosha et al. 2016; Hunduma and Ashenafi 2011).

The microbial population and thus the characteristics of kocho may vary over time and between different batches. Moreover, since in enset based agriculture in Ethiopia cattle manure is used as fertiliser, there might be hygienic problems with the food. Strict sanitation and hygiene procedures are not practiced during production, handling and processing of enset and its food products. However, not much is known about the microbiology of kocho. In spite of the great importance that enset-based food represents for the Ethiopian population, only few studies have been performed investigating the microbial flora during fermentation, as well as spoilage organisms. In most of these studies, identification was not done using molecular techniques, instead, less reliable physiological and morphological characteristics have been used for species identification, both for bacteria and yeasts. Yeasts were in many cases only identified to the genus level and frequently invalid taxonomic terms were used, making it impossible to draw any conclusion about the role of yeast species in kocho fermentation (Gashe 1987a, b; Gizaw et al. 2016; Tsegay et al. 2016). Very recently, Andeta et al. (2018), used 16S ribosomal RNA (rRNA) gene sequencing to identify bacteria from enset samples within the range of one to 60 days of fermentation period. Yeasts and moulds were quantified by cultivation on selective media but not identified.

It was our intention to isolate yeasts and bacteria from short (2–5 days)-, middle (3–4 months) - and long-period (7 months to 1 year) fermented enset materials and to identify these microbes by molecular methods, namely: 16S rDNA and D1D2 region of the 26S rDNA and/or ITS region to reliably identify bacteria and yeasts, respectively, to species level. By this we would obtain potentially beneficial microbes that may be used in starter cultures to make fermentations more predictable, increasing the nutritional value of the final product. Our approach will also identify potentially undesirable microorganisms that need to be controlled during fermentation. Thus, this study represents a step towards improved health and food safety of a considerable number of consumers.

## Materials and methods

*Sampling* Fresh samples were obtained from five farms of South-West Ethiopia (N: 7°44′38.148″, E: 35°28′34.3884′, height 2271 m above sea level; N: 8°5′11.94″, E: 37°59′20.04″ height 2392 m above sea level; N: 8°54′15.912″, E: 42°7′36.948″, height 1600 m above sea level; N: 9°1′59.304″, E: 38°45′0.288″, height 2449 m above sea level; N: 10°23′5.28″, E: 39°8′3.876″, height 2285 m above sea level): newly prepared kocho (2–5 days old, freshly decorticated and pulverised), medium time (kocho fermented for 3–4 months) and long-time fermented samples (fermented for 7 months up to 1 year) from farmers homestead plots, wrapped with layers of leaf sheets and buried in underground pits. Samples were packed in sterile Falcon tubes, placed on ice and transported to the laboratory. The materials from the different farms were mixed before extracting the microbes, to get a general survey about the microbes at the different fermentation times. About 10 g of the fermented material was suspended in 100 ml saline (9 g NaCl l^−1^) and appropriate dilutions were spread on three different selective growth media and incubated at 30 °C for one to 3 days, until colonies became visible. Selective media were yeast extract peptone dextrose (YPD) agar with chloramphenicol (100 mg l^−1^) for the isolation of yeasts, tryptone glucose extract agar (TGEA) with cycloheximide (500 mg l^−1^) for the isolation of general aerobic bacteria, and de Man, Rogosa and Sharp (MRS) Agar with delvocide (100 mg l^−1^) for the isolation of lactic acid bacteria (LABs) (Birmeta et al. 2004b). LAB were incubated in anaerobic conditions, using GasPak jars and a GasPak anaerobic (Becton–Dickinson, Sparks, MD, USA).

*Identification of Microorganisms* To ensure a random choice of isolates for identification, all colonies of a certain predefined area were identified, usually all colonies from an appropriate dilution plate with 10–20 colonies. The clones were grouped by a PCR-fingerprint as described earlier (Olstorpe et al. 2008). Representative clones were chosen for molecular identification by sequencing their D1D2/ITS region of the 26S rDNA or their 16S rDNA for yeasts or bacteria, respectively. The clones were identified by comparing the sequences with those of known strains using BLAST search of the EMBL-database.

*PCR and sequencing* Amplification and sequencing of the yeast and bacterial rDNA sequences was performed as described before, using ExTaq™ polymerase (Takara Bioinc, Japan) according to the provider’s recommendations (Birmeta et al. 2004b). PCR fingerprints were done using a GTG_5_-primer (Lieckfeldt et al. 1993). Reaction conditions for the fingerprints were 5 min 95 °C; 29 cycles of 30 s 94 °C, 30 s 50 °C, 2 min 72 °C; 6 min at 72 °C (final extension). Sequencing was done by the Molecular Cloning Laboratories (San Francisco, USA).

## Results and discussion

Microorganisms were extracted from kocho and spread onto different selective media as described in Materials and Methods. The number of viable microbes in the different samples ranged for yeasts 10^4^–10^6^ and for bacteria 10^7^–10^9^ per g material (wet weight), similarly as described by Andeta et al. (2018). Because it was our aim to provide a general survey about the species present in the material, we did not perform an exact quantification of the cfu-numbers of the different microbial groups in the different samples. Ten to twenty colonies from each extraction were randomly chosen for identification. From each of the colonies, DNA was extracted and PCR-fingerprints were generated. Based on the fingerprint patterns the isolates were divided in types. For one to two representatives of the different types, the rDNA stretches were amplified and sequenced. In case where two isolates of a fingerprint pattern were sequenced, they were identified as the same species. This shows that the fingerprint provided a satisfying resolution for identification.

In total, 29 different culturable species were identified. These include 12 yeast species, 6 LAB- species, and 11 aerobic bacteria (Tables 1, 2, 3). There were more yeast species identified in fresh cultures compared with long-term fermented kocho samples (Table 1). This may be an indication of decreasing diversity over time, however, further investigation and identification of more isolates is required to confirm this trend.

**Table 1:** Yeast species isolated and identified by sequencing D1 D2 and or ITS region of 26S rDNA from fresh, mid-term and long-term fermented kocho samples

Fermentation periods	Identified species	Number of isolates	Frequency (%)
Fresh (2–5 days)	*Pichia exigua*	5	25
	*Galactomyces geotrichum*	5	25
	*Pichia fermentans*	4	20
	*Saturnispora silvae*	2	10
	*Pichia barkeri/nakasei*	2	10
	*Wickerhamomyces pijperi*	1	5
	*Kazachstania exigua*	1	5
Total		20	100
Midterm (3–4 months)	*Galactomyces geotrichum*	9	36
	*Candida cabralensis*	10	40
	*Candida ethanolica*	4	16
	*Kregervanrija fluxuum*	1	4
	*Pichia membranifaciens*	1	4
Total		25	100
Long term (7–12 months)	*Saturnispora silvae*	9	75
	*Cyberlindnera jadinii*	2	17
	*Galactomyces geotrichum*	1	8
Total		12	100

**Table 2:** Lactic Acid Bacteria (LABs) isolated under anaerobic conditions and identified by sequencing 16S rDNA region from kocho samples obtained from fresh, mid-term and long-term fermented kocho samples

Fermentation periods	LAB identified	Number of isolates	Frequency (%)
Fresh (2–5 days)	*Lactobacillus plantarum*	20	95
	*Weissella cibaria*	1	5
Total		21	100
Midterm (3–4 months)	*Lactobacillus plantarum*	20	95
	*Weissella cibaria*	1	5
Total		21	100
Longterm (7–12 months)	*Acetobacter pasteurianus*	5	42
	*Lactobacillus plantarum*	3	25
	*Leuconostoc mesenteroides/pseudomesenteroides*	2	17
	*Acetobacter cerevisiae*	1	8
	*Lactobacillus buchneri*	1	8
Total		12	100

**Table 3:** Total aerobic bacteria isolated and identified by sequencing 16S rDNA from fresh and long-term fermented kocho samples

Fermentation periods	Bacteria identified	Number of isolates	Frequency (%)
Fresh (2–5 days fermented)	*Pantoea agglomerans*	9	30
	*Raoultella planticola*	12	40
	*Acinetobacter* sp.	3	10
	*Empedobacter* sp.	3	10
	*Citrobacter farmeri*	1	3
	*Leuconostoc citreum*	1	3
	*Enterobacter kobei*	1	3
Total		30	100
Long term (7–12 months)	*Bacillus simplex*	4	40
	*Bacillus anthracis*	3	30
	*Bacillus thuringiensis*	1	10
	*Lactobacillus plantarum*	1	10
	*Microbacterium* sp. SKJH-22	1	10
Total		10	100

In fresh kocho, seven different yeast species were identified; most frequently *Pichia exigua*, *Galactomyces geotrichum* (each 25%) and *Pichia fermentans* (20%). In midterm kocho, five yeast species were isolated and the most frequent yeasts were *Candida cabralensis* (40% of all isolates), *Galactomyces geotrichum* (36%) and *Candida ethanolica* (16%). *C. ethanolica* was finally distinguished from its sister species *Pichia deserticola* by sequencing the ITS-region (Wu and Bai 2005). *C. cabralensis* and *C. ethanolica* (iter. nom. *Pichia*) belong to the *Pichia* lineage (Daniel et al. 2014), nevertheless, no official re-naming has been published yet. In the long-term fermentations three yeast species were identified (Table 1). The most frequently isolated yeast (9 out of 12 isolates) was *Saturnispora silvae* (originally described as *Candida silvae*) (Kurtzman 2015; Vidal-Leiria and van Uden 1963). The other two species were *Cyberlindnera jadinii* (two isolates) and *G. geotrichum* (one isolate).

Our study for the first time identified yeasts from different fermentations stages of kocho by molecular methods. Gashe (1987a) quantified yeasts from kocho and identified them to the level of genera. However, these genera included e.g. *Torulopsis* and *Candida*, which are (in the first case) no longer taxonomically valid or (in both cases) polyphyletic categories, which are not informative about phylogenetic relationships. More recently, identification to species level of yeasts isolated from kocho was tried (Gizaw et al. 2016; Tsegay et al. 2016), unfortunately only based on non-reliable morphological and physiological characteristics. Moreover, invalid taxonomic designations (e.g. *Candida zylandase*) were used. This implies that the role of yeasts in kocho fermentation is still unclear. It has been hypothesised that yeasts may hydrolyse the starch to provide simple sugars for subsequent lactic acid fermentation (Andeta et al. 2018; Karssa et al. 2014). However, none of our identified yeasts is able to utilise starch, even maltose, the major disaccharide from starch degradation, is only assimilated by *C. jadinii*, which was isolated from the long- term fermented kocho (Table 1).

Interestingly, the yeast population was similar to those of some fermented dairy products. This may be due to the fact that kocho-producing farms are also running cattle and cheese production for own consumption or selling. Due to these dairy-related activities, microbes can be transferred from one system to the other. The yeasts *G. geotrichum*, which was found in all three fermentation stages, and *C. cabralensis*, which was quite abundant in mid-term fermented kocho have been both isolated from dairy products. *G. geotrichum* has been isolated from milk, cheeses, but also some starch-based alcoholic drinks in Southern Europe and China. Apart from assimilating sugars and lactic acid, the yeast is supposed to hydrolyse lipids and proteins and to inhibit the formation of biogenic amines, which has a positive influence on the taste of the final product (Grygier et al. 2017). *C. cabralensis* has been isolated from Spanish blue-veined Cabrales cheese and from Portugese Serpa cheese (Flórez et al. 2010; Gonçalves Dos Santos et al. 2017). In fact, our study is the first documented isolation of this yeast from another source than cheese. *S. silvae* was isolated from humans, horses, and marine invertebrates in a Brazilian mangrove ecosystem (de Araujo et al. 1995, Vidal-Leiria and van Uden 1963). Nevertheless, it was also frequently associated with fermented food, including dairy products and sourdoughs (e.g. Laurencik et al. 2008; Ongol and Asano 2009; Taccari et al. 2016). Similarly, *P. fermentans* has been isolated from a variety of environments, including fruit juices where it was supposed to be a spoilage organism (Las Heras-Vazquez et al. 2003). It was also found in the cereal-based beverage boza and in fermented pig feed. Both systems are rich in starch and contain considerable numbers of LAB (Caputo et al. 2012; Olstorpe et al. 2010), similar to kocho-fermentation. *P. exigua* was isolated from tarubá, a beverage produced by cassava fermentation, which is also very rich in LAB (Ramos et al. 2015). *C. ethanolica* has originally been isolated from fodder yeast production on ethanol (Rybářová et al. 1980), but also recently from tarubá-production (Ramos et al. 2015).

Most of the yeast isolates are none- or weak fermenters, i.e. they require oxygen for growth (Kurtzman et al. 2011). This is to some extent unexpected in terms of mid- and long-term fermented kocho, as the material is stored in underground pits, where one can expect oxygen limitation. Obviously, the practice of regularly opening the pits and mixing the material (Hunduma and Ashenafi 2011) provides enough oxygen for the yeasts to grow and survive. Many, if not all of the isolated yeasts can assimilate lactic acid (Kurtzman et al. 2011). This may indicate that they rather act as spoilage organisms, decreasing acidity by consuming lactic acid and thus decreasing conservation power of the fermented material, similar to silages (Wilkinson and Davies 2013). On the other hand, it has been shown that yeasts may release LAB-stimulating metabolites (Wyder et al. 1999).

Only two LAB species were found in fresh and midterm fermented kocho, with *Lactobacillus plantarum* being the most frequently isolated organism (95%) at both fermentation periods (Table 2). The other LAB-species found in fresh- and mid-term fermented kocho, *Weissella cibaria* has been frequently isolated from fermented foods, including those that are like kocho rich in starch, such as sourdough (Fusco et al. 2015). Five LAB species were found in the long-term fermented kocho, most frequently *Acetobacter pasteurianus* (42%), *L. plantarum* (25%) and *Leuconostoc mesenteroides/pseudomesenteroides* (17%). *L. mesenteroides* can only be distinguished from *L. pseudomesenteroides* by polygenic sequencing, because the 16S rRNA- genes are identical to more than 99.5% (Gu et al. 2012). In previous works, *Leuconostoc* spp. has frequently been found already at the beginning of kocho fermentation (Andeta et al. 2018; Gashe 1987a). Nevertheless, in the culture- independent identification by Andeta et al. (2018), its frequency varied between 10 and 30%. It is possible that the sensitivity of our assay was not high enough to detect this species in the initial fermentation stages. In the long-term fermented kocho, a high proportion of acetic acid bacteria was isolated on the LAB- selective media. Acetic acid bacteria can metabolise sugars, sugar alcohols and ethanol to acetic acid (Raspor and Goranovic 2008). They are acid tolerant and can obviously tolerate the lactic acid that is produced by the LAB. For kocho, longer fermentation time is associated with improved quality and taste of the final product (Hunduma and Ashenafi 2011). This may be due to a higher diversity of LAB and the presence of acetic acid bacteria in long-term fermented kocho, which can result in an increased number of metabolites, and thus, richness in taste and improved conservation.

To gain insight into hygienic and health aspects of the kocho fermentation, we also identified eight aerobic bacteria in fresh and five in long-term fermented kocho samples (Table 3). In fresh kocho, all isolates from fresh kocho, except *Leuconostoc citreum* were gram-negative. The most frequently identified species were *Raoultella planticola* (40%) and *Pantoea agglomerans* (30%). *R. planticola* (Drancourt et al. 2001) is a bacterium that is found in soil and on plants, occasionally also on humans and animals. It can assimilate starch (Hii et al. 2012), has been found in farms running dairy production and is an opportunistic pathogen (Sekowska 2017; Zadoks et al. 2011). *P. agglomerans* lives in a variety of habitats, including plants, arthropods or vertebrates. In plants it often acts as symbiont, but in some cases it can also be a pathogen. Cases where it acted as pathogen on humans have also been documented (Dutkiewicz et al. 2016).

In long-term fermented kocho, mainly spore forming bacteria, *Bacillus simplex* (40%) and *Bacillus anthracis* (30%) were found. The LAB *L. plantarum* was also identified on the general bacterial selective medium. The identification of *B. anthracis* represents a critical finding. This bacterium is a pathogen causing anthrax and, due to its ability to form spores, it is also very persistent. Anthrax is a zoonotic disease endemic to Africa. *Bacillus* species are common soil species, and they might be introduced into the material due to the practice of placing the material into a pit in the soil, poor hygiene and sanitation, and manure use. Due to their ability of spore formation they may survive low pH, microbial competition and shortage of nutrients in the final product. *B. anthracis* and *B. thuringiensis* both belong to the *Bacillus cereus* group, which is seen as a single species by a growing number of scientists. Toxicity and pathogenicity to animals and humans is related to the presence of virulence plasmids. Infection of humans mainly occurs upon contact with infected animals (Maughan and Van der Auwera 2011). Moreover, the presence of anaerobic spore forming bacteria (*Clostridia*) in kocho has also been demonstrated (Andeta et al. 2018; Gashe 1987a). In our study, we did not have the technical opportunity to test for *Clostridia*, but one can assume that they were present in the tested food material. The presence of spores or active cells of pathogens in food represents a risk to the consumers. However, to our knowledge kocho consumption has never been reported to cause human disease (Vieira et al. 2017). This might be due to a generally rather low number of bacteria in the final product and their inactivation during the subsequent baking process.

More studies are required to understand the interaction of all microbes in kocho-fermentation. Acidification of the fermented material requires the initial degradation of starch. As stated above, the identified yeast species cannot degrade starch, thus, this has to be performed either by endogenous enzymes similar to starch degradation in sourdough (De Vuyst et al. 2017) and/or by the bacteria present in the process. Many of the frequently identified bacteria in this study have been demonstrated to degrade starch. These include strains of *L. plantarum* and *L. mesenteroides*- amylolytic strains were frequently isolated from starchy fermented foods in tropical climate (Giraud et al. 1994; Johansson et al. 1995; Reddy et al. 2008), *R. planticola* (Hii et al. 2012), *P. agglomerans* (Costa et al. 2002) and *Bacillus* spp. (Halami 2008; Heyrman et al. 2005). Hunduma and Ashenafi (2011) isolated a variety of amylolytic bacteria from different kocho fermentations. The yeasts in the process may stimulate LAB and, together with LAB contribute to the final taste of the product (De Vuyst et al. 2017; Grygier et al. 2017; Wyder et al. 1999).

A predictable microbial population during the fermentation process is highly desirable, both in terms of obtaining a product of good taste and high nutritional value and preventing or at least decreasing the occurrence of potentially pathogenic organisms. Measures should be taken to improve the safety of the final product. Those measures should include more strict sanitation and hygiene (including education of farmers about ways of transferring microbes from their cattle production to the fermented food), and the development of appropriate starter cultures. Starter cultures can make the microbial population more predictable and can help to control undesirable pathogenic microbes in the fermentation process. LABs develop antimicrobial activities by a combination of mechanisms, including formation of organic acids and the bacteriocins (Ross et al. 2002). For instance, *L. plantarum* has been described to form plantaricin W (Holo et al. 2001). Yeasts have also been demonstrated to show activities against bacteria and fungi, due to competition for nutrients and oxygen, production of antimicrobial metabolites such as ethanol, organic acids or esters or killer proteins (Muccilli and Restuccia 2015; Olstorpe et al. 2012; Olstorpe and Passoth 2011). A starter culture containing both appropriate LABs and yeasts may provide an efficient barrier against pathogenic microbes by combining several different antimicrobial activities.

Our study provides the first survey of kocho-fermentation, where cultured isolates were identified by molecular methods. Culture-dependent methods have the disadvantage that they only monitor a part of the microbial population. On the other hand, they provide a survey about viable microbes in the fermentation, and isolates can serve as source for starter cultures. More studies with proper identification of involved microbes are required to understand the microbial interactions and metabolic pathways crucial for the sensory and nutritional quality of the fermented food.
